# Combination of gamma-glutamyl transferase and liver stiffness measurement for biliary atresia screening at different ages: a retrospective analysis of 282 infants

**DOI:** 10.1186/s12887-020-02172-z

**Published:** 2020-06-04

**Authors:** Qiulong Shen, Sarah Siyin Tan, Zengmeng Wang, Siyu Cai, Wenbo Pang, Chunhui Peng, Yajun Chen

**Affiliations:** 1 Department of General Surgery, Beijing Children’s Hospital, Capital Medical University, National Center for Children’s Health, No.56 Nanlishi St, Xicheng District, Beijing, 100045 China; 2 Center for Clinical Epidemiology and Evidence-based Medicine, Beijing Children’s Hospital, Capital Medical University, National Center for Children’s Health, No.56 Nanlishi St, Xicheng District, Beijing, 100045 China

**Keywords:** Biliary atresia, Infantile cholestatic jaundice, Misdiagnosis, Parallel test

## Abstract

**Background:**

This study aims to explore the diagnostic accuracy of the combination of gamma-glutamyl transferase (GGT) and liver stiffness measurement (LSM) for biliary atresia (BA) screening at different ages.

**Methods:**

Our retrospective study involved 282 infants under the age of 120 days with jaundice who were admitted into Beijing Children’s Hospital between January 2016 to December 2018. The GGT and LSM levels of infants were obtained. A parallel test was used, and ROC curve was created to obtain cutoff values of GGT and LSM for BA infants at different ages.

**Results:**

Of the 282 infants, 135 were diagnosed with BA and 147 were non-BA infants. In all age groups (A: ≤60 days; B: 61–90 days; C: 91–120 days), the LSM and GGT levels of the BA group were significantly higher than that of the non-BA group, *P* < 0.05. The cutoff value of GGT and LSM to diagnosis BA was 191.2 U/L, 213.2 U/L, 281.5 U/L and 7.5 kPa, 10.0 kPa, 11.0 kPa in groups A, B and C, respectively. The parallel test was used to determine a sensitivity of 97.3, 98.1 and 100% in group A, B and C when either GGT or LSM levels were met in BA infants. The sensitivities of parallel testing for group A and B were higher than LSM or GGT used alone.

**Conclusions:**

Cutoff values of GGT and LSM to screen BA increased with age. Parallel testing of GGT and LSM in infants who are younger than 90 days old can decrease the rate of BA misdiagnosis.

## Background

Biliary Atresia (BA) is a common cause for infants with infantile cholestatic jaundice. Early diagnosis and surgical treatment have been demonstrated to be of great importance for better prognosis [1]. Hepatic fibrosis, liver failure and death can result from untreated BA. Currently, the accepted method for diagnosing BA is intraoperative cholangiography. Due to non-specific clinical features, there is no preoperative investigation that allows a definite diagnosis of BA [2].

Both gamma-glutamyl transferase (GGT) and liver stiffness measurement (LSM) are tests which aid in increasing the likelihood of an infant having BA [3, 4]. GGT measures the proliferation of bile ducts, which is a pathological presentation in BA [5]. LSM is used to measure the level of liver fibrosis [6]. Progressive fibroinflammatory cholangiopathy is the main presentation of BA and leads to hepatic fibrosis. The severity of hepatic fibrosis increases with the age of the infant. Since infants of different ages have different degrees of hepatic pathological injury, a single cut-off value for LSM and GGT might create a diagnosing bias.

This study explored the diagnostic accuracy of the combination of GGT and LSM in different ages for the diagnosis of BA, in order to reduce the missed diagnosis rate and achieve early diagnosis.

## Methods

### Patients

This retrospective study involved 282 infants with jaundice who were admitted into Beijing Children’s Hospital of Capital Medical University between January 2016 to December 2018. Inclusion criteria were: 1) Age ≤ 120 days, 2) Direct bilirubin > 1 mg/dl, 3) documentation of preoperative GGT and LSM results. Infants who had non-BA related surgical history that led to jaundice were excluded. Infants were further divided into 3 groups according to their age, group A (≤60 days), group B (61–90 days), group C (91–120 days). Age was the age at which the LSM was completed. This study has been approved by the Ethical Committees of Beijing Children’s Hospital.

### Data collection

Patient’s liver functions were collected, included GGT, alkaline phosphatase (ALP), aspartate aminotransferase (AST), alanine aminotransferase (ALT), total bilirubin (TIBL) and direct bilirubin (DBIL), which were measured on the same day as LSM.

### LSM measurement

LSM was performed with an Aixplorer ultrasound system (SuperSonic Imagine SA, Aix-en-Provence, France) and an L15–4 linear probe. The measurements were spilt among 2 physicians and analyzed. The inter−/intraobserver variability was not assessed. Both physicians had more than 5 years of experience in abdominal sonography. The site of insonation approximated the surgical biopsy site at the right lower hepatic lobe. LSM was targeted at liver parenchyma free of large vessels, with the upper edge 0.5 to 1 cm away from the liver capsule. The region of interest for LSM was 1.0 cm diameter. The mean values of 5 consecutive measurements were used for statistical analyses, and IQR/median < 0.3 was used as a quality criterion. Our team started obtaining LSM with this machine for BA children in 2016. Previously, when LSM was measured, Fibroscan was used. In order to ensure the reliability of our data, we choose January 2016 to December 2018 as our study period.

### Statistical analysis

Statistical analysis was performed using SPSS18.0. Two independent sample T-test was used to analyze statistical differences between the two groups for quantitative data that was consistent with normal distribution, and expressed as mean ± SD. The remaining data was analyzed using rank sum test and expressed using median and interquartile range. *P* < 0.05 was considered significant. Correlation analysis and multivariate logistic regression analysis were used to analyze the correlation. The receiver operating characteristic (ROC) curve analysis was used to determine the final cutoff value. Chi-square test was used to compare the difference in effectiveness of GGT and LSM. Parallel test was used to calculate sensitivity levels of GGT and LSM.

## Results

### Patient characteristics

Two hundred eighty-two infants with infantile cholestatic jaundice (171 males, 111 females) were enrolled in the study, among whom 135 infants (65 males, 70 females) were in the BA group and 147 infants were in the non-BA group (106 males, 41 females). All infants in the BA group were classified as type III BA during their Kasai surgery. Infants in the non-BA group were diagnosed with other illnesses that can also cause infantile cholestatic jaundice. These include idiopathic cholestasis (*n* = 67, 45.6%), hereditary metabolic liver disease (*n* = 37, 25.2%), cytomegalovirus hepatitis (*n* = 36, 24.5%), progressive familial intrahepatic cholestasis (PFIC) (*n* = 6, 4.1%) and Alagille syndrome (*n* = 1, 0.6%).

**Fig. 1:**
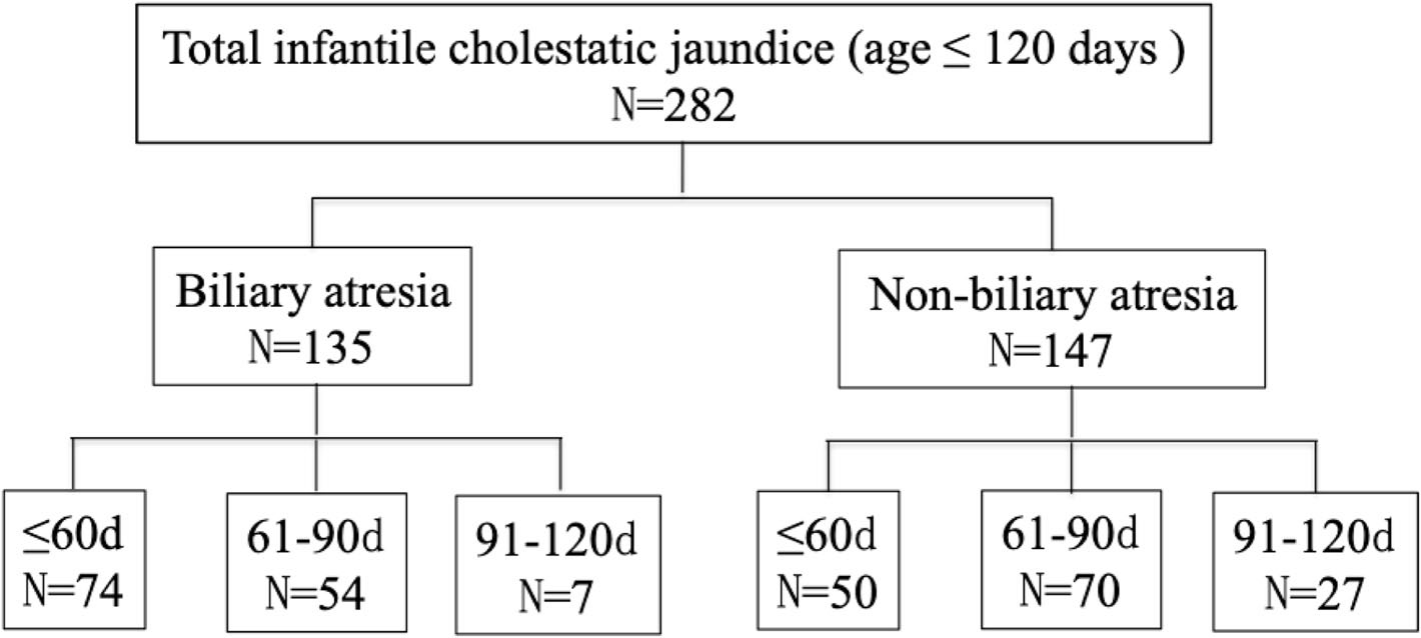
Flow chart of subject enrollment

In the BA group, there were 74, 54 and 7 infants in groups A, B and C respectively. In the non-BA group, there were 50, 70 and 27 infants in groups A, B and C, respectively (Fig.1). All patients included in the study were 25 days old or older. Infants younger than 30 days old (9 cases) were not grouped separately due to the small number of infants in that age group.

### Correlation between age, GGT and LSM

**Fig.2:**
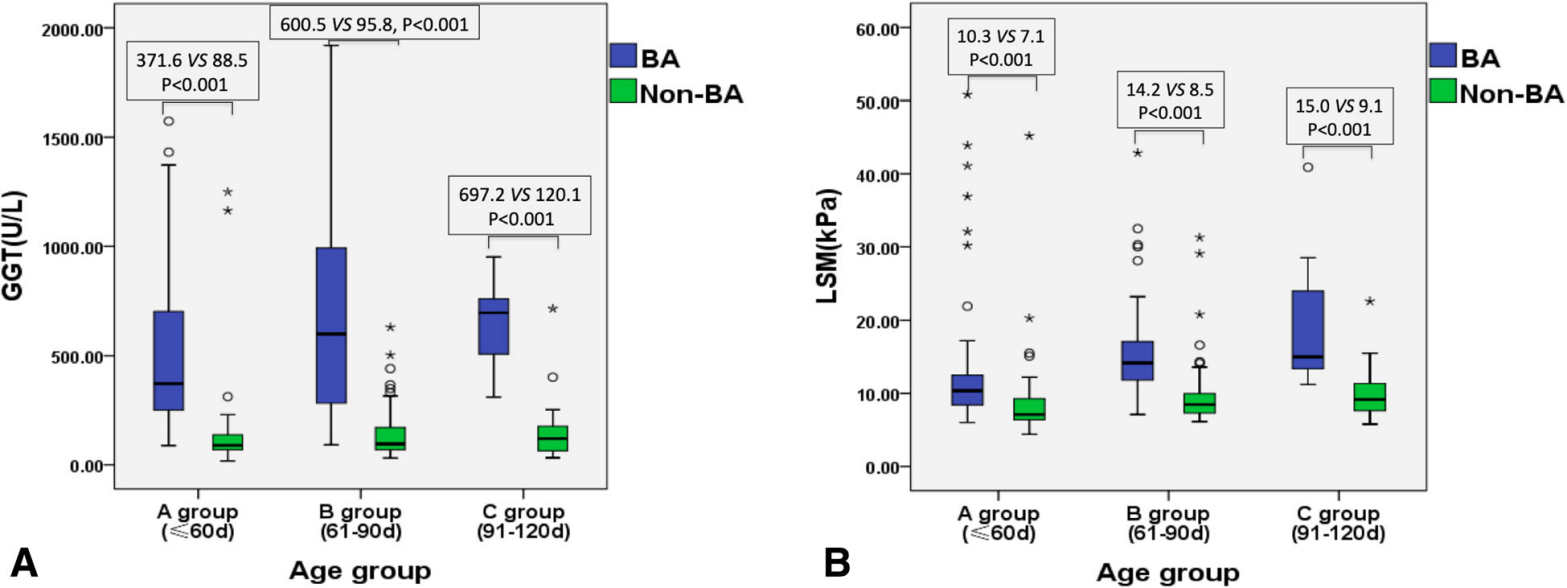
**a**. Box plot of GGT in different age groups. **b**. Box plot of LSM in different age groups

The age of infants in the BA group (59 ± 18.8 days) was younger than that of the non-BA group (70 ± 20.4 days), *P* < 0.001. In all age groups, the LSM and GGT levels of the BA group was significantly higher than that of the non-BA group, *P* < 0.05 (Table 1, Fig. 2).

**Table 1:** The LSM and GGT of infants in the BA and non-BA group

		BA group	Non-BA group	*P* value
All patient	N	135	147	–
	LSM, kPa	12.0(6.0)	8.1(3.3)	< 0.001
	GGT, U/L	455.3(556.4)	95.0(96.0)	< 0.001
Group A (≤60d)	N	74	50	–
	LSM, kPa	10.3(4.2)	7.1(3.0)	< 0.001
	GGT, U/L	371.6(458.4)	88.5(72.1)	< 0.001
Group B (61-90d)	N	54	70	–
	LSM, kPa	14.2(5.4)	8.5(2.8)	< 0.001
	GGT, U/L	600.5(722.7)	95.8(103.4)	< 0.001
Group C (91-120d)	N	7	27	–
	LSM, kPa	15.0(16.4)	9.1(4.3)	< 0.001
	GGT, U/L	697.2(429.6)	120.1(122.7)	< 0.001

*LSM* Liver stiffness measurement, *GGT* Gamma-glutamyl transferase

The data was analyzed using rank sum test and expressed using median and interquartile range (IQR)

There was statistical significance for LSM between groups A, B and C in both BA and non-BA infants (*P* < 0.01). In the BA group, the difference in GGT in groups A, B and C showed a statistical significance (*P* = 0.044), but this was not demonstrated in the non-BA group (*P* = 0.697).

### Correlation of GGT and LSM with other factors

There was no significant correlation between GGT and alkaline phosphatase (ALP), aspartate aminotransferase (AST), alanine aminotransferase (ALT), total bilirubin (TIBL) and direct bilirubin (DBIL) in the BA group (*P* > 0.05), but there was a significant correlation between LSM and both AST and ALT (*P* < 0.01). The correlation between GGT and AST, ALT, DBIL in the non-BA group was statistically significant (*P* < 0.05), and the correlation between LSM and ALP, AST, ALT, TIBL and DBIL was also statistically significant (*P* < 0.01) (Table 2).

**Table 2:** The correlation of GGT and LSM with other factors

	GGT		LSM	
	*r* value (BA/non-BA)	*P* value (BA/non-BA)	*r* value (BA/non-BA)	*P* value (BA/non-BA)
ALP (U/L)	−0.113/0.044	0.192/0.600	0.011/0.290	0.896/< 0.001
AST (U/L)	−0.066/− 0.277	0.449/0.001	0.331/0.504	< 0.001/< 0.001
ALT (U/L)	−0.046/− 0.364	0.592/< 0.001	0.250/0.372	0.003/< 0.001
TBIL (umol/l)	−0.077/− 0.151	0.375/0.068	0.113/0.389	0.194/< 0.001
DBIL (umol/l)	0.026/−0.180	0.765/0.030	0.103/0.422	0.234/< 0.001

*LSM* Liver stiffness measurement, *GGT* Gamma-glutamyl transferase, *ALP* Alkaline phosphatase, *AST* Aspartate aminotransferase, *ALT* Alanine aminotransferase, *TBIL* Total bilirubin, *DBIL* Direct bilirubin

Multivariate logistic regression analysis with adjustments for several covariates including GGT, LSM, ALP, ALT, AST, TBIL and DBIL were performed, and showed that there were independent differences in GGT and LSM between BA and non-BA group.

### ROC analysis for GGT

**Fig. 3:**
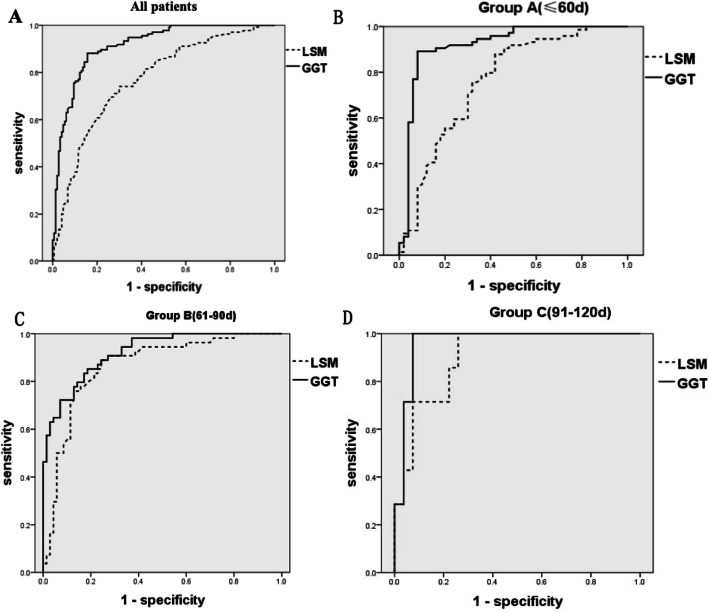
ROC curves of LSM and GGT in all patients (**a**), group A (**b**), group B (**c**) and group C (**d**)

ROC curve analysis showed a GGT cutoff value of 192.5 U/L was optimal for predicting BA, the area under the ROC curve (AUC) was 0.914, sensitivity 88.9%, specificity 85.0%, positive predictive value (PPV) 84.5%, negative predictive value (NPV) 89.3%. The ROC curve analysis of different age groups showed that the optimal cutoff value was 191.2 U/L (Groups A: AUC 0.919, sensitivity 89.2%, specificity 92.0%), 213.2 U/L (Groups B: AUC 0.921, sensitivity 85.2%, specificity 81.4%), 281.5 U/L (Groups C: AUC 0.963, sensitivity 100%, specificity 92.6%), respectively (Table 3, Fig. 3).

**Table 3:** The ROC curve analysis results of GGT and LSM

		AUC	Youden index	95% CI	Cut-off value	sensitivity	specificity	PPV	NPV
All patients	GGT, U/L	0.914	0.739	0.881–0.947	192.5	0.889	0.850	0.845	0.893
	LSM, kPa	0.771	0.434	0.715–0.825	9.5	0.733	0.701	0.692	0.741
Group A (≤60d)	GGT, U/L	0.919	0.812	0.861–0.977	191.2	0.892	0.920	0.943	0.852
	LSM, kPa	0.761	0.458	0.671–0.850	7.5	0.878	0.580	0.756	0.763
Group B (61-90d)	GGT, U/L	0.921	0.666	0.877–0.966	213.2	0.852	0.814	0.797	0.892
	LSM, kPa	0.865	0.646	0.798–0.932	10.0	0.889	0.757	0.738	0.898
Group C (91-120d)	GGT, U/L	0.963	0.926	0.903–1.000	281.5	1.000	0.926	0.778	1.000
	LSM, kPa	0.905	0.741	0.800–1.000	11.0	1.000	0.741	0.636	1.000

*CI* Confidence interval, *PPV* Positive predictive value, *NPV* Negative predictive value, *LSM* Liver stiffness measurement, *GGT* Gamma-glutamyl transferase, *ROC* The receiver operating characteristic, *AUC* The area under the ROC curve

### ROC analysis for LSM

ROC curve analysis showed a LSM cutoff value of 9.5 kPa was optimal for predicting BA, AUC 0.771, sensitivity 73.3%, specificity 70.1%, PPV 69.2%, NPV 74.1%. The ROC curve analysis of different age groups showed that the optimal cutoff value was 7.5 kPa (Group A: AUC 0.761, sensitivity 87.8%, specificity 58.0%), 10.0 kPa (Group B: AUC 0.865, sensitivity 88.9%, specificity 75.7%), 11.0 kPa (Group C: AUC 0.905, sensitivity 100%, specificity 74.1%), respectively (Table 3, Fig. 3).

### Comparison of LSM and GGT

Chi-square test showed a statistical difference between LSM and GGT when used to distinguish BA and non-BA infants (*P* = 0.011) in group A. GGT was a better indicator for BA. There was no statistical difference in group B (*P* = 0.418). The chi-square test could not be performed for group C due to the small number of infants.

### Parallel test for GGT and LSM

**Fig. 4:**
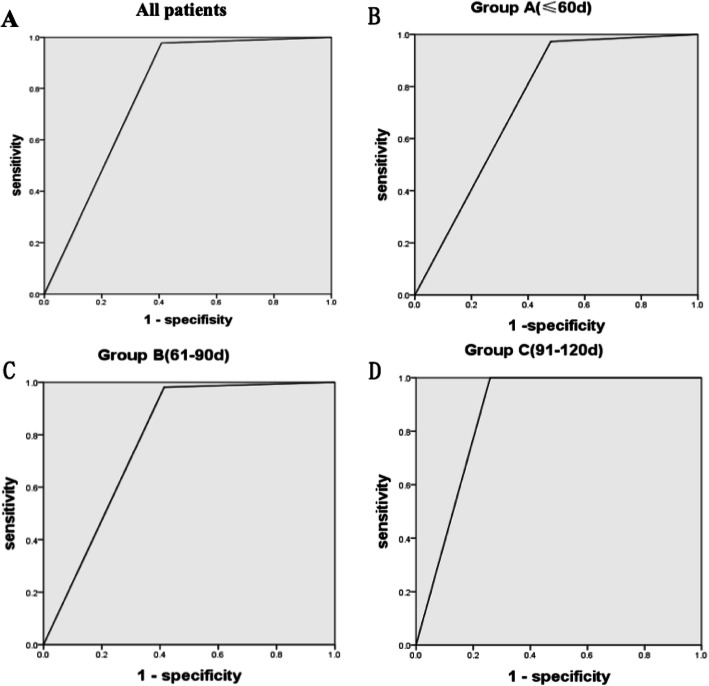
ROC curves of parallel test in all patients (**a**), group A (**b**), group B (**c**) and group C (**d**)

The parallel test was used to determine a sensitivity of 96.3% when GGT > 192.5 U/L or LSM > 9.5 kPa was used to diagnose BA (specificity was 58.8%, PPV 68.1%, NPV 94.5%). This was a higher sensitivity level than either LSM (73.3%) or GGT (88.9%) alone. The sensitivity level of group A and B was 97.3 and 98.1% respectively, which was also higher than LSM or GGT alone. In group C, the sensitivity level of LSM and GGT was already 100%, similar to the parallel test (Table 4, Fig. 4). Chi-square test was used to compare the diagnostic effectiveness between (1) GGT and the parallel test, and (2) LSM and the parallel test, and it was found that both were statistically significant (*P* < 0.001).

**Table 4:** The parallel test results for GGT and LSM in BA infants

		AUC	Youden index	95% CI	sensitivity	specificity	PPV	NPV
All patients	GGT > 192.5 U/L or LSM > 9.5 kPa	0.785	0.570	0.730–0.840	0.978	0.592	0.681	0.945
Group A (≤60d)	GGT > 191.2 U/L or LSM > 7.5 kPa	0.746	0.493	0.651–0.842	0.973	0.520	0.735	0.923
Group B (61-90d)	GGT > 213.2 U/L or LSM > 10.0 kPa	0.784	0.567	0.703–0.865	0.981	0.586	0.654	0.976
Group C (91-120d)	GGT > 281.5 U/L or LSM > 11.0 kPa	0.870	0.741	0.753–0.988	1.000	0.741	0.583	1.000

*CI* Confidence interval, *PPV* Positive predictive value, *NPV* Negative predictive value, *LSM* Liver stiffness measurement, *GGT* Gamma-glutamyl transferase, *AUC* The area under the receiver operating characteristic curve

## Discussion

There are various causes of infantile cholestatic jaundice including biliary or chromosomal abnormalities, infectious diseases, idiopathic cholestasis and others. Among them, the most common cause is BA, and accounts for 20–40% of infantile cholestatic jaundice [7]. BA is a hepatobiliary disease that occurs in infancy and is characterized by progressive fibroinflammatory obliterative cholangiopathy, which can ultimately lead to liver cirrhosis if left untreated [8]. It is imperative to differentiate BA from other diseases that also cause infantile cholestatic jaundice at an early age because progress of hepatic fibrosis occurs quickly. The mainstay of treatment is the Kasai surgery [9], and age at surgery is most consistently correlated with clinical outcome, with younger patients having a better native liver survival [1]. This demonstrates the importance of early-stage BA screening for patients with infantile cholestatic jaundice. The aim of our paper was to use a parallel test to aid in the screening of children with infantile cholestatic jaundice who have BA.

The diseases included in this study that need to be differentiated from BA included idiopathic cholestasis, hereditary metabolic liver disease, cytomegalovirus hepatitis, PFIC and Alagille syndrome. Our ultimate goal is for other basic centers to use our findings to screen BA, and if patients are found to be positive for the parallel test, we recommend that they should be transferred to a specialized children’s hospital for further testing. PFIC patients usually have low-GGT levels but because they can also present with infantile cholestatic jaundice, we felt that it was more reasonable to include PFIC cases in the study.

Currently, the accepted method for diagnosing BA is intraoperative cholangiography, but this is an invasive test, and is not suitable for widespread usage in infantile cholestatic jaundice. The main non-invasive tests include ultrasound, LSM and liver function laboratory tests [10–13].

Ultrasound is the most common examination used for infantile cholestatic jaundice. The typical ultrasound presentation of BA includes gallbladder abnormalities and triangular cord sign at the hepatic hilar region. A 2015 meta-analysis found that sensitivity and specificity for gallbladder abnormalities was 85 and 92%, compared to 74 and 97% for triangular cord sign [14]. However, ultrasound diagnosis is very subjective, and accuracy depends on the competency of individual physicians.

Lately, studies have established the relationship between elevated levels of GGT and BA. It is now widely acknowledged that GGT can aid in the diagnosis of BA. When used to diagnose BA, El-Guindi et al. used a cutoff of GGT > 286 U/L and found sensitivity and specificity levels to be 76.7 and 80% respectively [15]. Comparatively, our cut-off value was GGT > 192.5 U/L and our sensitivity and specificity levels were 88.9 and 85.0%. In addition, this study showed that GGT levels were not affected by other liver function measurements in the BA group. It was only in the non-BA group that GGT demonstrated a slight correlation with AST and ALT. With the exception of GGT, liver function laboratory tests are generally used as part of a scoring equation to help indicate the degree of liver fibrosis for BA infants and is rarely used for directly diagnosing BA.

The use of LSM has gradually been applied to diagnosis of BA infants. Wu et al. found that LSM > 7.7 kPa had a sensitivity of 80% and specificity of 97% [4]. In this study, our cut-off value was LSM > 9.5, and our sensitivity and specificity levels were 73.3 and 70.1%. A possible reason for the low sensitivity and specificity of LSM could be that LSM is affected by biochemical indicators. The results of this study showed that the LSM in the non-BA group were significantly affected, and a positive correlation existed, ultimately leading to LSM being higher than its corresponding liver fibrosis grade.

Although not yet a main non-invasive test for BA, serum matrix metalloproteinase-7 (MMP-7) has a high sensitivity and specificity level (98.6 and 95.0%). However, it requires a specific enzyme-linked immunosorbent assay and thus is currently unable to be routinely used in hospitals [10]. Among non-invasive tests, GGT and LSM are more objective [16], as they can be easily performed and repeated.

GGT is widely recognized as an indicator to distinguish BA from other infantile cholestatic jaundice infants [17]. GGT is mainly found in intrahepatic bile duct endothelial cells and in the cytoplasm of hepatocytes. When there is obstruction of the intra and extra hepatic bile duct, excretion is blocked, causing a backflow and resulting in an increase in serum GGT. An increase in GGT can also reflect hyperplasia or inflammation of liver bile duct inflammation. The main characteristic pathological presentation of BA is the proliferation of bile ducts [5], which causes serum GGT in BA infants to be higher than that of non-BA infants. In BA patients, pathological changes of the liver become more severe with age, the level of proliferation increases, and GGT levels also demonstrate a similar trend. In non-BA patients, an increase in GGT levels is due to obstruction of the bile ducts and not proliferation, thus their GGT levels do not have a correlation with age. This coincides with the findings of the study. We found that the cutoff value for GGT levels increase with age and presents with a higher sensitivity and specificity level. This finding was also replicated in Xiaoli Chen’s study [18]. However, it should be noted that the study of Xiaoli Chen et al. observed a decrease in GGT after 120 days of age. They proposed that after 120 days, biliary endothelial cells are seriously damaged and therefore no longer have the ability to produce GGT.

Compared to infants with other diseases leading to infantile cholestatic jaundice, BA’s main pathological characteristic is progressive fibrosis of the liver. LSM can determine the degree of liver fibrosis, and research suggests that LSM is a good preoperative diagnosis tool for BA [19, 20]. However, the number of infants in such studies are limited. Wu’s study comprised of 48 infantile cholestatic jaundice cases, of whom 15 were BA infants at the age of 27–60.5 days and had a cutoff value of 7.7 kPa [4]. Comparatively, our study had 124 infants who were 25–60 days old, of whom 74 were diagnosed with BA and had an LSM cutoff value of 7.5 kPa. Both cutoff values for LSM were similar, but Wu’s sensitivity and specificity levels were higher than our results. Nonetheless, we are relatively confident that our study has a better representation of the general population as the higher number of infants provide a more reliable result. With an increase in age, the severity of liver fibrosis becomes greater, which leads to an increase of LSM. Our study showed that the cutoff value of LSM increased with age. This reinforces the need for early diagnosis of BA infants within infantile cholestatic jaundice.

In group A, the ability of GGT to diagnose BA infants was better than LSM. This advantage was not demonstrated in group B. Although GGT and LSM were both equally useful, their sensitivity level did not reach 90%. Since misdiagnosis of BA can lead to devasting outcomes, we used the parallel test to raise sensitivity levels, thereby reducing the rate of misdiagnosis. However, parallel testing will cause a decrease in specificity levels, and should only be used as a screening method. If patients are found to be positive for the parallel test, we recommend that they be transferred to a specialized children’s hospital for further testing. The sensitivity level for group A and group B was 79.3 and 98.1% respectively, which was markedly higher than that of LSM or GGT alone. Both the parallel test and individual tests for Group C showed a sensitivity level of 100%. We recommend that infants under the age of 90 days with infantile cholestatic jaundice should undergo this parallel test to screen for BA. This will greatly decrease the rate of misdiagnosis. For infants who are older than 90 days old, either GGT or LSM can be used to screen for BA.

There are some shortcomings in this paper. During the study period, we advocated for our center to use LSM and GGT in infants who present with infantile cholestatic jaundice. This practice did indeed lead to quicker diagnosis of BA and the age of infants at time of surgery to be younger. However, we were unable to calculate the data about the accurate decreasing number of misdiagnoses which could further support our conclusion, because most of the patients in this study were referred by other hospitals, but not all hospitals have LSM examination equipment. If there is no LSM examination equipment in the hospital and it is not convenient to go to other hospitals for examination, clinical judgment must come into play according to comprehensive information from GGT, ultrasound, etc. If BA is suspected, it is recommended that the child be transferred to a hospital which specializes in pediatric surgery.

## Conclusions

In conclusion, cutoff values of GGT and LSM to screen BA increased with age. Parallel testing of GGT and LSM in infants who are younger than 90 days old can decrease the rate of BA misdiagnosis, thus reducing the age at time of Kasai operation and ultimately improving the prognosis of BA.

## Data Availability

The data is available from the corresponding author on reasonable request.

## Abbreviations

BA: Biliary atresia
LSM: Liver stiffness measurement
GGT: r-glutamyl transferase
ALP: Alkaline phosphatase
AST: Aspartate aminotransferase
ALT: Alanine aminotransferase
TBIL: Total bilirubin
DBIL: Direct bilirubin
ROC: Receiver operating characteristic
AUROC: Area under the receiver operating characteristic
